# Supplementary material for: Analysis of anti-SARS-CoV-2 Omicron-neutralizing antibody titers in different vaccinated and unvaccinated convalescent plasma sources
Source: Nat Commun. 2022 Oct 29;13:6478. doi: 10.1038/s41467-022-33864-y (PMC9617541; doi:10.1038/s41467-022-33864-y)
Supplement: Supplementary file 1 — Suplementary Information [file 41467_2022_33864_MOESM1_ESM.pdf]

# Supplementary Information

Supplementary Information ........ 1
Supplementary table 1 ........ 2
Supplementary table 2 ........ 4
Supplementary table 3 ........ 5
Supplementary table 4 ........ 6
Supplementary table 5 ........ 7
Supplementary table 6 ........ 9
Supplementary table 7 ........ 10
Supplementary table 8 ........ 12
Supplementary table 9 ........ 14
Supplementary table 10 ........ 16
Supplementary table 11 ........ 18
Supplementary table 12 ........ 20
Supplementary Figure 1 ........ 21
Supplementary Figure 2 ........ 22
Supplementary Figure 3 ........ 23
Supplementary Figure 4 ........ 24
Supplementary Figure 5 ........ 25
Supplementary Figure 6 ........ 26
Supplementary Figure 7 ........ 27
Supplementary Figure 8 ........ 28
Supplementary Figure 9 ........ 29
Supplementary Figure 10 ........ 30
Supplementary Figure 11 ........ 31
References ........ 32

# Supplementary table 1

Synopsis of 27 *in vitro* studies investigating the efficacy of pre-Alpha CCP against Omicron

| Reference | Assay type (live virus =1 Pseudovirus=2) | Time mean or median (min-max) | (pre-Alpha CCP) WA-1 $GMT_{50}$ | (pre-Alpha CCP) BA.1 $GMT_{50}$ | (pre-Alpha CCP) fold drop vs. BA.1 | (pre-Alpha CCP) number in study | (pre-Alpha CCP) BA.1 neutralizing number | (pre-Alpha CCP) BA.1 neutralizing percent |
|---|---|---|---|---|---|---|---|---|
| Zeng[1] | 2 | (1-2) mo | 1712 | 14 | 122 | 9 | 1 | 11 |
| Liu[2] | 2 | (9-120) days | 4344 | 135 | 32 | 10 | 2 | 20 |
| Schmidt[3] | 2 | 1.2 mo | 2616 | 68 | 38 | 20 | 19 | 95 |
| Schmidt[3] | 2 | 12 mo | 2037 | 136 | 15 | 20 | 17 | 85 |
| Schmidt[3] | 2 | 6 mo | 1678 | 34 | 49 | 20 | 13 | 65 |
| Arien[4] | 1 | 1 mo | 1086 | 50 | 22 | 10 | 1 | 10 |
| Lusvarghi[5] | 2 | 30 (14-51) days | 715 | 25 | 29 | 16 | 2 | 13 |
| Hoffman[6] | 2 | < 2 mo | 614 | 8 | 77 | 17 | 8 | 47 |
| Zou[7] | 1 | 1 mo | 601 | 38 | 16 | 64 | 41 | 64 |
| Planas[8] | 2 | 6 mo | 569 | 28 | 20 | 16 | 6 | 38 |
| Planas[8] | 2 | 12 mo | 580 | 29 | 20 | 23 | 8 | 35 |
| Zhang[9] | 2 | (1-3) mo | 556 | 66 | 8 | 28 | 28 | 100 |
| Gruell[10] | 2 | 1.5 mo | 494 | 6 | 82 | 30 | 3 | 10 |
| Gruell[10] | 2 | 12 mo | 93 | 8 | 12 | 30 | 9 | 30 |
| Dejnirattisai[11] | 1 | 42 med days | 475 | 28 | 17 | 32 | 32 | 100 |
| Sheward[12] | 2 | 1 mo | 350 | 40 | 9 | 34 | 25 | 74 |

| | | | | | | | | |
|---|---|---|---|---|---|---|---|---|
| Tada[13] | 2 | (32-57) | 233 | 9 | 26 | 10 | 4 | 40 |
| Aggerwal[14] | 1 | 1+ mo | 215 | 10 | 22 | 20 | 0 | 0 |
| Zhao[15] | 2 | 18 (11-51) days | 193 | 11 | 17 | 16 | 1 | 6 |
| Bowen[16] | 2 | (28-78) days | 162 | 10 | 16 | 28 | 13 | 46 |
| Zou[7] | 1 | 6 mo | 142 | 32 | 4 | 36 | 30 | 83 |
| Carreno[17] | 1 | 58 (23-87) days | 67 | 6 | 11 | 15 | 4 | 27 |
| Syed[18] | 2 | <1 mo | 80 | 20 | 4 | 8 | 6 | 75 |
| Bekliz[19] | 1 | (3-137) days | 37 | 1 | 45 | 34 | 5 | 15 |
| Haveri[20] | 1 | 1 mo | 32 | 2 | 16 | 13 | 0 | 0 |
| LI[21] | 1 | <1 mo | 28 | 2 | 14 | 108 | 22 | 22 |
| Kurahashi[22] | 1 | (1-3) mo | 19 | 2 | 13 | 40 | 15 | 38 |
| GM (GMT$_{50}$) | | | 1712 | 15 | 20 | | | |
| Total (AVG%) | | | | | | 707 | 315 | (45%) |

# Supplementary table 2

Synopsis of 5 *in vitro* studies investigating the efficacy of Alpha CCP against Omicron

| Reference | Assay type (live virus =1 Pseudovirus=2) | Time mean or median (min-max) | (Alpha CCP) WA-1 $GMT_{50}$ | (Alpha CCP) BA.1 $GMT_{50}$ | (Alpha CCP) fold reduction vs. BA.1 | (Alpha CCP) number | (Alpha CCP) BA.1 neutralizing number | (Alpha CCP) BA.1 neutralizing percent |
|---|---|---|---|---|---|---|---|---|
| Lusvarghi[5] | 2 | 30 (14-51) days | 4978 | 30 | 166 | 4 | 1 | 25 |
| Dejnirattisai[11] | 1 | 19 med days | 1313 | 39 | 34 | 18 | 18 | 100 |
| Rossler[23] | 1 | | 256 | 4 | 63 | 10 | 0 | 0 |
| Haveri[20] | 1 | 1 mo | 32 | 2 | 16 | 20 | 0 | 0 |
| Bekliz[19] | 1 | 3-137 days | 46 | 1 | 57 | 12 | 2 | 17 |
| GM ($GMT_{50}$) | | | 301 | 6 | 50 | | | |
| Total (AVG%) | | | | | | 64 | 21 | (33%) |

# Supplementary table 3

Synopsis of 5 *in vitro* studies investigating the efficacy of Beta CCP against Omicron.

| reference | Assay type (live virus =1 Pseudovirus=2) | time mean or median (min-max) | (beta CCP) WA-1 $GMT_{50}$ | (beta CCP) BA.1 $GMT_{50}$ | (beta CCP) fold reduction vs. BA.1 | (beta CCP) number | (beta CCP) BA.1 neutralizing number | (beta CCP) BA.1 neutralizing percent |
|---|---|---|---|---|---|---|---|---|
| Lusvarghi[5] | 2 | 30 (14-51) days | 439 | 191 | 2 | 2 | 2 | 100 |
| Dejnirattisai[11] | 1 | 61 med days | 327 | 28 | 12 | 14 | 14 | 100 |
| Rossler[23] | 1 | | 128 | 4 | 32 | 8 | 1 | 13 |
| Haveri[20] | 1 | 1 mo | 16 | 2 | 8 | 5 | 0 | 0 |
| Bekliz[19] | 1 | 3-137 days | 21 | 1 | 23 | 8 | 2 | 25 |
| GM ($GMT_{50}$) | | | 140 | 8 | 11 | | | |
| Total (AVG%) | | | | | | 37 | 19 | (51%) |

# Supplementary table 4

Synopsis of 7 *in vitro* studies investigating the efficacy of Delta CCP against Omicron.

| Reference | Assay type (live virus =1 Pseudovirus=2) | Time mean or median (min-max) | (Delta CCP) WA-1 $GMT_{50}$ | (Delta CCP) BA.1 $GMT_{50}$ | (Delta CCP) fold drop vs. BA.1 | (Delta CCP) number | (Delta CCP) BA.1 neutralizing number | (Delta CCP) BA.1 neutralizing percent |
|---|---|---|---|---|---|---|---|---|
| Zeng[1] | 2 | (1-2) mo | 11200 | 3476 | 3 | 19 | 10 | 53 |
| Lechmere[24] | 2 | (13-22) days | 668 | 164 | 4 | 14 | 12 | 86 |
| Lusvarghi[5] | 2 | 30 (14-51) days | 1211 | 18 | 66 | 15 | 12 | 80 |
| Aggerwal[14] | 1 | | 770 | 37 | 21 | 10 | 9 | 90 |
| Rossler[23] | 1 | | 192 | 8 | 25 | 7 | 1 | 14 |
| Bekliz[19] | 1 | 3-137 days | 73 | 3 | 24 | 10 | 6 | 60 |
| Dejnirattisai[11] | 1 | 38 med dayas | 47 | 27 | 2 | 19 | 19 | 100 |
| GM ($GMT_{50}$) | | | 167 | 10 | 17 | | | |
| Total (AVG%) | | | | | | 94 | 69 | (73%) |

# Supplementary table 5

Synopsis of 22 *in vitro* studies investigating the efficacy of plasma from uninfected recipients of 2 BNT162b2 doses against Omicron.

| reference | Assay type (live virus =1 Pseudo virus=2) | time mean or median (min-max) | (2 dose BNT162 b2 plasma) WA-1 $GMT_{50}$ | (2 dose BNT162 b2 plasma) BA.1 $GMT_{50}$ | (2 dose BNT162 b2 plasma) fold reduction vs. BA.1 | (2 dose BNT162 b2 plasma) number | (2 dose BNT162b 2 plasma) BA.1 neutralizing number | (2 dose BNT162b 2 plasma) BA.1 neutralizing percent |
|---|---|---|---|---|---|---|---|---|
| Schmidt[3] | 2 | 1 mo | 7627 | 92 | 83 | 18 | 15 | 83 |
| Liu[2] | 2 | (16-213) days | 4669 | 222 | 21 | 13 | 6 | 46 |
| Zeng[1] | 2 | (21-28) days | 2769 | 121 | 23 | 48 | 13 | 27 |
| Schmidt[3] | 2 | 5 mo | 2435 | 126 | 19 | 18 | 15 | 83 |
| Dejnirattisai[11] | 1 | 1 mo | 1993 | 19 | 105 | 20 | 20 | 100 |
| Chatterjee[25] | 2 | 4 mo | 1544 | 935 | 2 | 25 | 25 | 100 |
| Syed[18] | 2 | 1-1.5 mo | 1280 | 80 | 16 | 21 | 14 | 67 |
| Tada[13] | 2 | 90 days | 859 | 25 | 34 | 9 | 7 | 78 |
| Bowen[16] | 2 | 9-20 days | 764 | 28 | 27 | 10 | 9 | 90 |
| Chatterjee[25] | 2 | 1 mo | 641 | 105 | 6 | 19 | 10 | 53 |
| Hoffman[6] | 2 | <3 mo | 604 | 10 | 60 | 11 | 1 | 9 |
| Lusvarghi[5] | 2 | 30 (28-34) days | 562 | 22 | 26 | 39 | 3 | 8 |
| Gruell[10] | 2 | 1 mo | 546 | 8 | 68 | 30 | 10 | 33 |
| Rossler[23] | 1 | 1 mo | 512 | 24 | 21 | 20 | 9 | 45 |
| Edara[26] | 1 | (14-28) days | 384 | 20 | 19 | 13 | 2 | 15 |

| | | | | | | | | |
|---|---|---|---|---|---|---|---|---|
| Muik[27] | 1 | 1 mo | 368 | 6 | 61 | 25 | 8 | 32 |
| Cele[28] | 1 | (10-48) days | 263 | 12 | 22 | 8 | 5 | 83 |
| Bekliz[19] | 1 | 1 mo | 338 | 4 | 86 | 16 | 11 | 69 |
| Planas[8] | 2 | 161 (138-176) days | 329 | 30 | 11 | 16 | 1 | 6 |
| Carreno[17] | 1 | 18 (14-21) days | 274 | 11 | 25 | 10 | 7 | 70 |
| Gruell[10] | 2 | 5 mo | 139 | 9 | 15 | 30 | 11 | 37 |
| Wilheim[29] | 1 | 6 mo | 6 | 1 | 6 | 15 | 0 | 0 |
| GM ($GMT_{50}$) | | | 628 | 27 | 24 | | | |
| Total (AVG%) | | | | | | 432 | 202 | (47%) |

# Supplementary table 6

Synopsis of 9 *in vitro* studies investigating the efficacy of plasma from uninfected recipients of 2 mRNA-1273 doses against Omicron.

| Reference | Assay type (live virus =1 Pseudovirus=2) | Time mean or median (min-max) | (2 dose mRNA-1273 plasma) WA-1 $GMT_{50}$ | (2 dose mRNA-1273 plasma) BA.1 $GMT_{50}$ | (2 dose mRNA-1273 plasma) fold drop vs. BA.1 | (2 dose mRNA-1273 plasma) number | (2 dose mRNA-1273 plasma) BA.1 neutralizing number | (2 dose mRNA-1273 plasma) BA.1 neutralizing percent |
|---|---|---|---|---|---|---|---|---|
| Doria-Rose[30] | 2 | 1 mo | 3016 | 62 | 49 | 30 | 22 | 73 |
| Syed[18] | 2 | 1-1.5 mo | 2560 | 320 | 8 | 10 | 8 | 80 |
| Doria-Rose[30] | 2 | 1 mo | 2269 | 27 | 84 | 30 | 22 | 73 |
| Bowen[16] | 2 | 6-15 days | 1155 | 26 | 44 | 11 | 9 | 82 |
| Tada[13] | 2 | 80 days | 999 | 38 | 26 | 8 | 5 | 63 |
| Edara[26] | 1 | 1 mo | 745 | 15 | 50 | 11 | 4 | 36 |
| Carreno[17] | 1 | 26 (14-36) days | 581 | 14 | 42 | 10 | 10 | 100 |
| Rossler[23] | 1 | 5 mo | 384 | 8 | 48 | 10 | 1 | 10 |
| Wilheim[29] | 1 | 6 mo | 10 | 1 | 20 | 14 | 0 | 0 |
| GM ($GMT_{50}$) | | | 644 | 21 | 31 | | | |
| Total (AVG%) | | | | | | 134 | 81 | (60%) |

# Supplementary table 7

Synopsis of 17 *in vitro* studies investigating the efficacy of plasma from infected and vaccinated (2 BNT162b2 doses) subjects (Vax-CCP) against Omicron.

| Reference | Assay type (live virus =1 Pseudovirus=2) | Time mean or median (min-max) | (post-COVID-19/full vacc plasma) WA-1 $GMT_{50}$ | (post-COVID-19/full vacc plasma) BA.1 $GMT_{50}$ | (post-COVID-19/full vacc plasma) fold drop vs. BA.1 | (post-COVID-19/full vacc plasma) number | (post-COVID-19/full vacc plasma) BA.1 neutralizing number | (post-COVID-19/full vacc plasma) BA.1 neutralizing percent |
|---|---|---|---|---|---|---|---|---|
| Schmidt[3] | 2 | 1 mo | 388872 | 8106 | 48 | 17 | 17 | 100 |
| Planas[8] | 2 | 32 (21-48) days | 78162 | 1475 | 53 | 22 | 22 | 100 |
| Tada[13] | 2 | 1 mo | 14868 | 921 | 16 | 7 | 7 | 100 |
| Cele[28] | 1 | (18-63) | 6763 | 305 | 22 | 13 | 13 | 100 |
| Kawoaka[31] | 1 | 1 mo | 10002 | 2029 | 5 | 13 | 13 | 100 |
| Kawoaka[31] | 1 | 3 mo | 2251 | 399 | 6 | 13 | 13 | 100 |
| Gruell[10] | 2 | 1.5 mo | 7997 | 1599 | 5 | 30 | 30 | 100 |
| Arien[4] | 1 | | 4822 | 236 | 20 | 10 | 10 | 100 |
| Carreno[17] | 1 | 26 (15-39) days | 2000 | 146 | 14 | 10 | 10 | 100 |
| Dejnirattisai[11] | 1 | 1 mo | 1899 | 215 | 9 | 17 | 17 | 100 |
| LI[21] | 1 | < 1 mo | 1598 | 80 | 20 | 20 | 20 | 100 |
| Bekliz[19] | 1 | 1 mo | 1190 | 65 | 18 | 6 | 6 | 100 |
| Haveri[20] | 1 | 1 mo | 1024 | 32 | 32 | 33 | 33 | 100 |
| Rossler[23] | 1 | | 1024 | 256 | 4 | 5 | 5 | 100 |
| Edara[26] | 1 | 6 mo | 625 | 31 | 20 | 24 | 15 | 63 |

| | | | | | | | | |
|---|---|---|---|---|---|---|---|---|
| Kurahashi[22] | 1 | 1 mo | 21 | 2 | 14 | 19 | 7 | 37 |
| Wilheim[29] | 1 | (.07-7.6) mo | 100 | 10 | 10 | 20 | 5 | 25 |
| GM ($GMT_{50}$) | | | 2753 | 192 | 14 | | | |
| Total (AVG%) | | | | | | 279 | 243 | (87%) |

# Supplementary table 8

Synopsis of 17 *in vitro* studies investigating the efficacy of plasma from uninfected subjects vaccinated with 3 BNT162b2 doses against Omicron.

| Reference | Assay type (live virus =1 Pseudovirus=2) | Time mean or median (min-max) | (3 dose BNT162b2 plasma) WA-1 $GMT_{50}$ | (3 dose BNT162b2 plasma) BA.1 $GMT_{50}$ | (3 dose BNT162b2 plasma) fold drop vs. BA.1 | (3 dose BNT162b2 plasma) number | (3 dose BNT162b2 plasma) BA.1 neutralizing number | (3 dose BNT162b2 plasma) BA.1 neutralizing percent |
|---|---|---|---|---|---|---|---|---|
| Schmidt[3] | 2 | 1 mo | 65617 | 3871 | 17 | 18 | 18 | 100 |
| Planas[8] | 2 | 33 (8-61) days | 12739 | 722 | 18 | 20 | 20 | 100 |
| Zeng[1] | 2 | (21-28) days | 10412 | 3179 | 3 | 23 | 20 | 87 |
| Dejnirattisai[11] | 1 | 1 mo | 9219 | 649 | 14 | 20 | 20 | 100 |
| Gruell[10] | 2 | 1 mo | 6241 | 1248 | 5 | 30 | 30 | 100 |
| Lusvarghi[5] | 2 | 43 (7-93) days | 5029 | 718 | 7 | 39 | 39 | 100 |
| Tada[13] | 2 | 1 mo | 4892 | 360 | 14 | 12 | 12 | 100 |
| Liu[2] | 2 | (14-90) days | 4673 | 715 | 7 | 15 | 15 | 100 |
| Kawoaka[31] | 1 | 1 mo | 2866 | 485 | 6 | 10 | 10 | 100 |
| Arien[4] | 1 | | 2157 | 165 | 13 | 10 | 10 | 100 |
| Hoffman[6] | 2 | <1 mo | 2006 | 305 | 7 | 10 | 9 | 90 |
| Edara[26] | 1 | (]7-28) days | 1247 | 89 | 14 | 35 | 31 | 89 |

| | | | | | | | | |
|---|---|---|---|---|---|---|---|---|
| Carreno[17] | 1 | 19 (14-28) days | 710 | 94 | 8 | 10 | 10 | 100 |
| Syed[18] | 2 | 1-1.5 mo | 960 | 160 | 6 | 8 | 8 | 100 |
| Muik[27] | 1 | 1 mo | 673 | 106 | 6 | 28 | 27 | 96 |
| Haveri[20] | 1 | (21–42) days | 256 | 16 | 16 | 7 | 7 | 100 |
| Wilheim[29] | 1 | 3 mo | 100 | 10 | 10 | 15 | 0 | 0 |
| GM ($GMT_{50}$) | | time mean or median (min-max) | 2588 | 290 | 9 | | | |
| Total (AVG%) | | | | | | 310 | 286 | (92%) |

59

# Supplementary table 9

Synopsis of *in vitro* studies investigating the efficacy of plasma from uninfected subjects vaccinated with 3 doses of mRNA-1273, AZD-1222 or Ad26.COV2 against BA.1. Because of diversity of vaccines the geomeans and sums were not computed.

| Reference | Vaccine type | WA-1 $GMT_{50}$ | Fold drop vs. BA.1 | BA.1 $GMT_{50}$ | Number | BA.1 neutralizing number | BA.1 neutralizing percent |
|---|---|---|---|---|---|---|---|
| Careno[17] | COVID19 + mRNA-1273 | 3000 | 11 | 272 | 10 | 10 | 100 |
| Edara[26] | COVID19 + mRNA-1273 6 mo | 931 | 25 | 38 | 13 | 9 | 69 |
| Careno[17] | 3 dose mRNA-1273 | 1000 | 17 | 60 | 10 | 10 | 100 |
| Doria-Rose[30] | 3 dose mRNA-1273 | 8457 | 4 | 2002 | 30 | 30 | 100 |
| Doria-Rose[30] | 3 dose mRNA-1273 | 4216 | 6 | 650 | 30 | 30 | 100 |
| Edara[26] | 3 dose mRNA-1273 | 1395 | 15 | 96 | 17 | 16 | 94 |
| Dejnirattisai[32] | AZD1222 | 390 | 19 | 21 | 41 | 41 | 100 |
| Rossler[23] | AZD1222 | 250 | 25 | 10.0 | 20 | 0 | 0 |
| Planas[8] | AZD1222 5 mo | 187 | 18 | 10 | 18 | 2 | 10 |
| Syed[18] | Ad26.COV2 | 28 | 1 | 20.0 | 9 | 2 | 22 |
| Schmidt[3] | Ad26.COV2 1 mo | 588 | 24 | 25 | 19 | 2 | 11 |

| | | | | | | | |
|---|---|---|---|---|---|---|---|
| Schmidt[3] | Ad26.COV2 6 mo | 982 | 23 | 43 | 19 | 11 | 58 |

# Supplementary table 10

Synopsis of *in vitro* studies investigating the efficacy of plasma from (pre-omicron, BA.1 and BA.4/5 CCP) subjects against Omicron BA.1, BA.2 and BA.4/5.

| Reference | Assay type (live virus =1 Pseudovirus =2) | Time mean or median (min-max) | WA-1 nAb $GMT_{50}$ | Omicron BA.1 nAb $GMT_{50}$ (Fold reduction from WA-1) | Omicron BA.2 nAb $GMT_{50}$ (Fold reduction from WA-1) | Omicron BA.14/5 nAb $GMT_{50}$ (Fold reduction from WA-1) | Number in study | BA.1; BA.2; BA.4/5 neutralizing number | BA.1; BA.2; BA.4/5 neutralizing percent |
|---|---|---|---|---|---|---|---|---|---|
| Preomicron | | | | | | | | | |
| Wang[33] | 1 | (7-213) days | 5185 | 845 (6 FR) | 939 (6 FR) | 596 (9 FR) | 22 | 22; 22;22 | 100;100 ; 100 |
| Awasthi[34] | 2 | >1 mo | 130 | 13 (10 FR) | 12 (11 FR) | 13 (13 FR) | 10 | 1; 1; 1 | 10; 10; 10 |
| Qu[35] | 2 | 3 day post hosp. | 3554 | 214 (17 FR) | 205 | 717 (5 FR) | 18 | 12; 14; 17 | 67; 78; 94 |
| GM ($GMT_{50}$) | | | 1338 | 133 (10 FR) | 132 (10 FR) | 177 (8 FR) | | | |
| Total (AVG%) | | | | | | | 50 | 35; 37;40 | (70;74; 80) |
| BA.1 | | | | | | | | | |
| Khan[36] | 1 | 23 (19-27iqr) | Not done | 275 | Not done | 36 | 24 | 24; nd; 18 | 100;nd; 75 |
| Zou[7] | 1 | 25 (13-62) days | 16 | 445 (0.04 FR) | 107 (0.15 FR | Not done | 20 | 20; 19; nd | 100;95; nd |

| | | | | | | | | | |
|---|---|---|---|---|---|---|---|---|---|
| Qu[35] | 2 | 1-8 days post hosp. | 317 | 399 (0.8 FR) | 304 (1.04 FR) | 189 (2 FR) | 30 | 22; 21; 23 | 73;70;77 |
| GM ($GMT_{50}$) | | | 71 | 366 (0.16 FR) | 180 (0.4 FR) | 82 (1 FR) | 74 | | |
| Total (AVG%) | | | | | | | | 66;40;41 | 89;80;93 |
| BA.4/5 | | | | | | | | | |
| Richardson[37] | 2 | < 9 days into hosp. | 904 | 557 (2 FR) | 884 (1 FR) | 1047 (1 FR) | 13 | 13;13;13 | 100;100; 100 |

69

# Supplementary table 11

Synopsis of *in vitro* studies investigating the efficacy of plasma from (post-COVID-19-BA.1/full vacc plasma) subjects (Vax-CCP) against Omicron BA.1, BA.2 and BA.4/5.

| reference | Assay type (live virus =1 Pseudovirus=2) | time mean or median (min-max) | WA-1 nAb $GMT_{50}$ | Omicron BA.1 nAb $GMT_{50}$ (Fold reduction from WA-1) | Omicron BA.2 nAb $GMT_{50}$ (Fold reduction from WA-1) | Omicron BA.14/5 nAb $GMT_{50}$ (Fold reduction from WA-1) | Number in study | BA.1; BA.2; BA.4/5 neutralizing number | BA.1; BA.2; BA.4/5 neutralizing percent |
|---|---|---|---|---|---|---|---|---|---|
| Muik[38] (BA.1) | 1 | 43 (25-55) | 780 | 656 (1 FR) | 800 (1 FR) | 156 (5 FR) | 14 | 14;14;13 | 100;100;93 |
| Muik[38] (BA.2) | 1 | 39 (30-99) | 531 | 347 (2 FR) | 545 (1 FR) | 209 (3 FR) | 13 | 13;13;13 | 100;100;100 |
| Khan[36] | 1 | | 2038 | 507 (4 FR) | | 158 (13 FR) | 15 | 15;nd;15 | 100;nd;100 |
| Wang [33] (BA.1) | 1 | (14-135) days | 9500 | 2406 (4 FR) | 2052 (5 FR) | 473 (20 FR) | 13 | 13;13;13 | 100;100;100 |
| Wang[33] (BA.2) | 1 | (14-36) days | 9527 | 3054 (3 FR) | 3679 (3 FR) | 1627 (6 FR) | 12 | 12;12;12 | 100;100;100 |
| Planas[39] | 1 | 80 med days | 10000 | 7000 (1 FR) | 9000 (1 FR) | 1000 (10 R) | 11 | 11;11;11 | 100;100;100 |
| Awasthi[34] | 2 | >1 mo | 1120 | 733 (2 FR) | 380 (3 FR) | 76 (15 FR) | 6 | 6;6;6 | 100;100;100 |
| Yu[40] | 2 | 14 med days | 4046 | 3249 (1 FR) | 2448 (2 FR) | | 8 | 7;7;nd | 88;88;nd |
| Richardson[37] | 2 | < 9 days into hosp. | 5484 | 4244 (1 FR) | 3779 (1 FR) | 1984 (3 FR) | 9 | 9;9;9 | 100;100;100 |

| | | | | | | | | | |
|---|---|---|---|---|---|---|---|---|---|
| Tuekprakhon [41] | 2 | 45 med days | 5861 | 3563 (2 FR) | 2119 (3 FR) | 1064 (6 FR) | 14 | 14;14;14 | 100;100; 100 |
| Hachman[42] | 2 | 29 (2-113) days | 11050 | 1740 (6 FR) | 1910 (6 FR) | 590 (19 (FR) | 27 | 27;27;27 | 100;100; 100 |
| GM ($GMT_{50}$) | | | 3578 | 1713 (2 FR) | 1830 ( 2 FR) | 454 (8 FR) | | | |
| Total (AVG%) | | | | | | | 142 | 141;126; 133 | 99;99;99 |

# Supplementary table 12

Synopsis of *in vitro* studies investigating the efficacy of plasma from (3 dose BNT162b2 plasma) subjects against Omicron BA.1, BA.2 and BA.4/5.

| reference | Assay type (live virus =1 Pseudovirus= 2) | time mean or median (min-max) | WA-1 nAb $GMT_{50}$ | Omicron BA.1 nAb $GMT_{50}$ (Fold reduction from WA-1) | Omicron BA.2 nAb $GMT_{50}$ (Fold reduction from WA-1) | Omicron BA.14/5 nAb $GMT_{50}$ (Fold reduction from WA-1) | Number in study | BA.1; BA.2; BA.4/5 neutralizing number | BA.1; BA.2; BA.4/5 neutralizing percent |
|---|---|---|---|---|---|---|---|---|---|
| Planas[39] | 1 | 132 med days | 2000 | 140 (14 FR) | 100 (20 FR) | 50 (40 FR) | 22 | 19;20;19 | 86;91;86 |
| Muik[38] | 1 | 28 (26-30) | 549 | 90 (6 FR) | 93 (6 FR) | 37 (15 FR) | 18 | 17;18; 17 | 94;100;94 |
| Khan[36] | 1 | | 4123 | 208 (20 FR) | Not done | 211 (20 FR) | 18 | 18;nd;;18 | 100;nd; 100 |
| Wang[33] | 1 | (14-90) days | 6657 | 130 (5 FR)0 | 1461 (5 FR) | 347 (19 FR) | 16 | 16;16;14 | 100;100; 88 |
| Qu[35] | 2 | (21-28) days | 2633 | 976 (3 FR) | 933 (3 FR) | 647 (4 FR) | 15 | 14;14;15 | 93;93; 100 |
| Tuekprakhon[41] | 2 | 28 days | 4122 | 1116 (4 FR) | 1113 (4 FR) | 360 (11 FR) | 19 | 19;19;19 | 100;100; 100 |
| Hachman[42] | 2 | 14 days | 5783 | 900 (6 FR) | 829 (7 FR) | 275 (21 FR) | 27 | 27;27;27 | 100;100; 100 |
| Yu[40] | 2 | 14 days | 6539 | 1066 (6 FR) | 776 (8 FR) | Not done | 24 | 24;24 | 100;100; nd |
| GM ($GMT_{50}$) | | | 3247 | 494 (7 FR) | 511 (6 FR) | 189 (16 FR) | 1 | | |
| Total (AVG%) | | | | | | | 159 | 154;138;129 | 97;98;96 |

# Supplementary Figure 1

Alternative graphing of Supplementary table 1 and Figure 2A. 27 *in vitro* studies investigating the efficacy of pre-Alpha CCP against Omicron with $GMT_{50}$ from each study with minimum and maximum dilution titer also shown. The absolute values are graphed in the upper panels for WA-1 on left and omicron on right. In the lower panels all numbers divided by the minimum value for each study to normalize for WA-1 on left and Omicron on right. The $GMT_{50}$ approximates 10 with this computational division. Live virus assays are on the left and pseudoviral assays on the right. Individual studies did not report $GMT_{50}$ standard deviation.

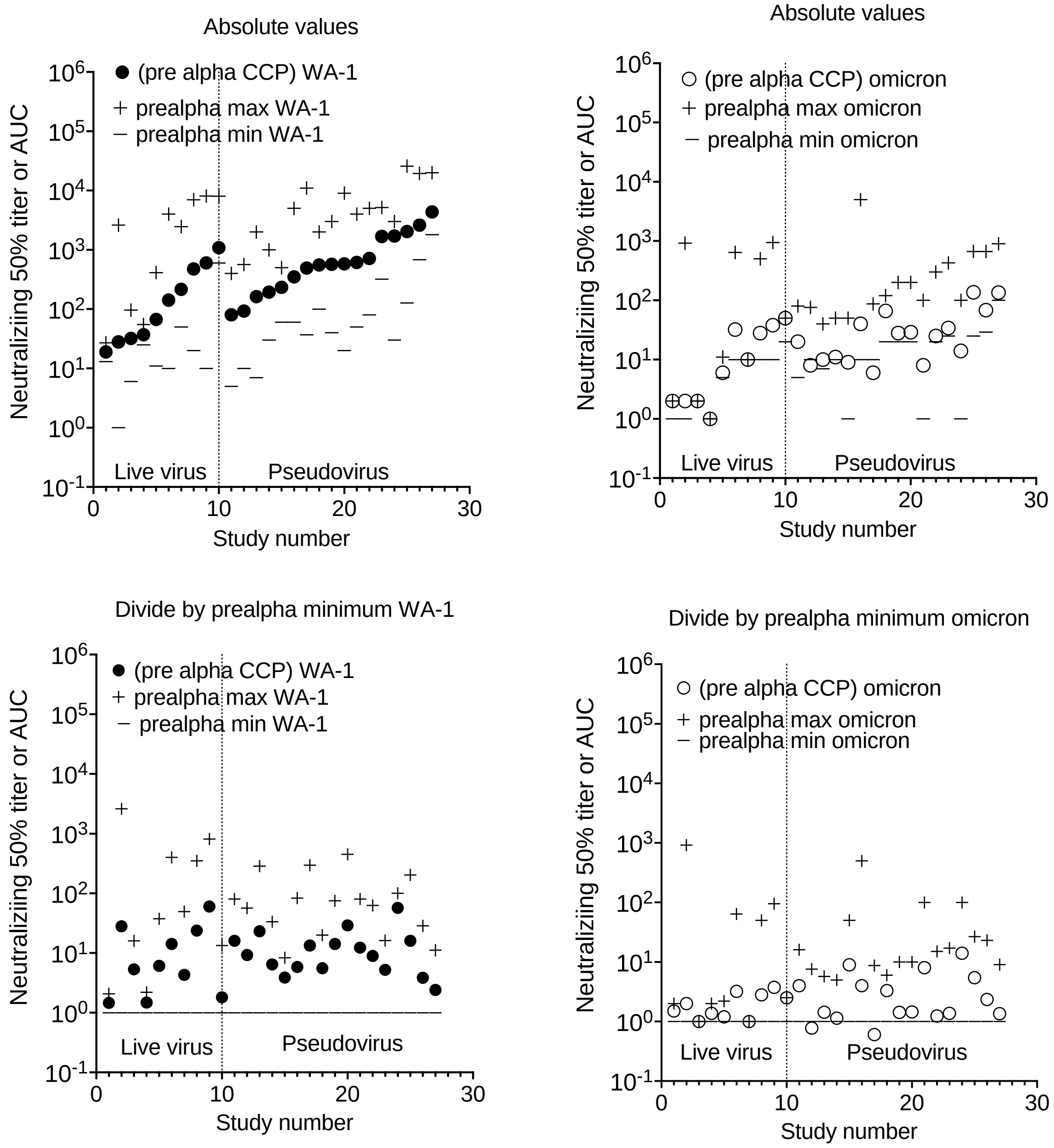

# Supplementary Figure 2

Alternative graphing of Supplementary table 2 and Figure 2A. 5 *in vitro* studies investigating the efficacy of Alpha CCP against Omicron with $GMT_{50}$ from each study with minimum and maximum dilution titer also shown. The absolute values are graphed in the upper panels for WA-1 on left and omicron on right. In the lower panels all numbers divided by the minimum value for each study to normalize for WA-1 on left and Omicron on right. The $GMT_{50}$ approximates 10 with this computational division. Live virus assays are on the left and pseudoviral assays on the right. Individual studies did not report $GMT_{50}$ standard deviation.

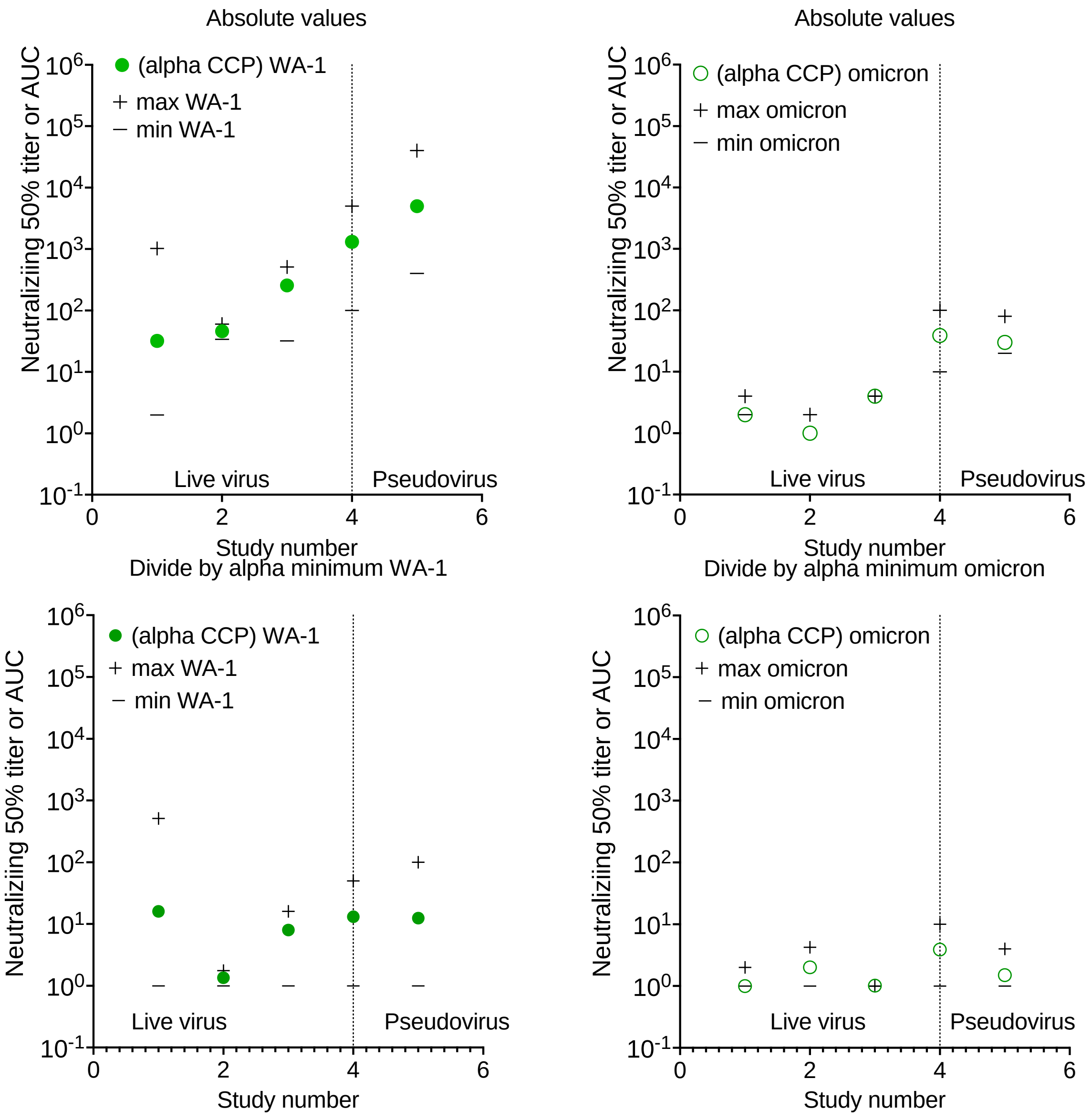

# Supplementary Figure 3

Alternative graphing of Supplementary table 3 and Figure 2A. 5 *in vitro* studies investigating the efficacy of Beta CCP against Omicron with $GMT_{50}$ from each study with minimum and maximum dilution titer also shown. The absolute values are graphed in the upper panels for WA-1 on left and omicron on right. In the lower panels all numbers divided by the minimum value for each study to normalize for WA-1 on left and Omicron on right. The $GMT_{50}$ approximates 10 with this computational division. Live virus assays are on the left and pseudoviral assays on the right. Individual studies did not report $GMT_{50}$ standard deviation.

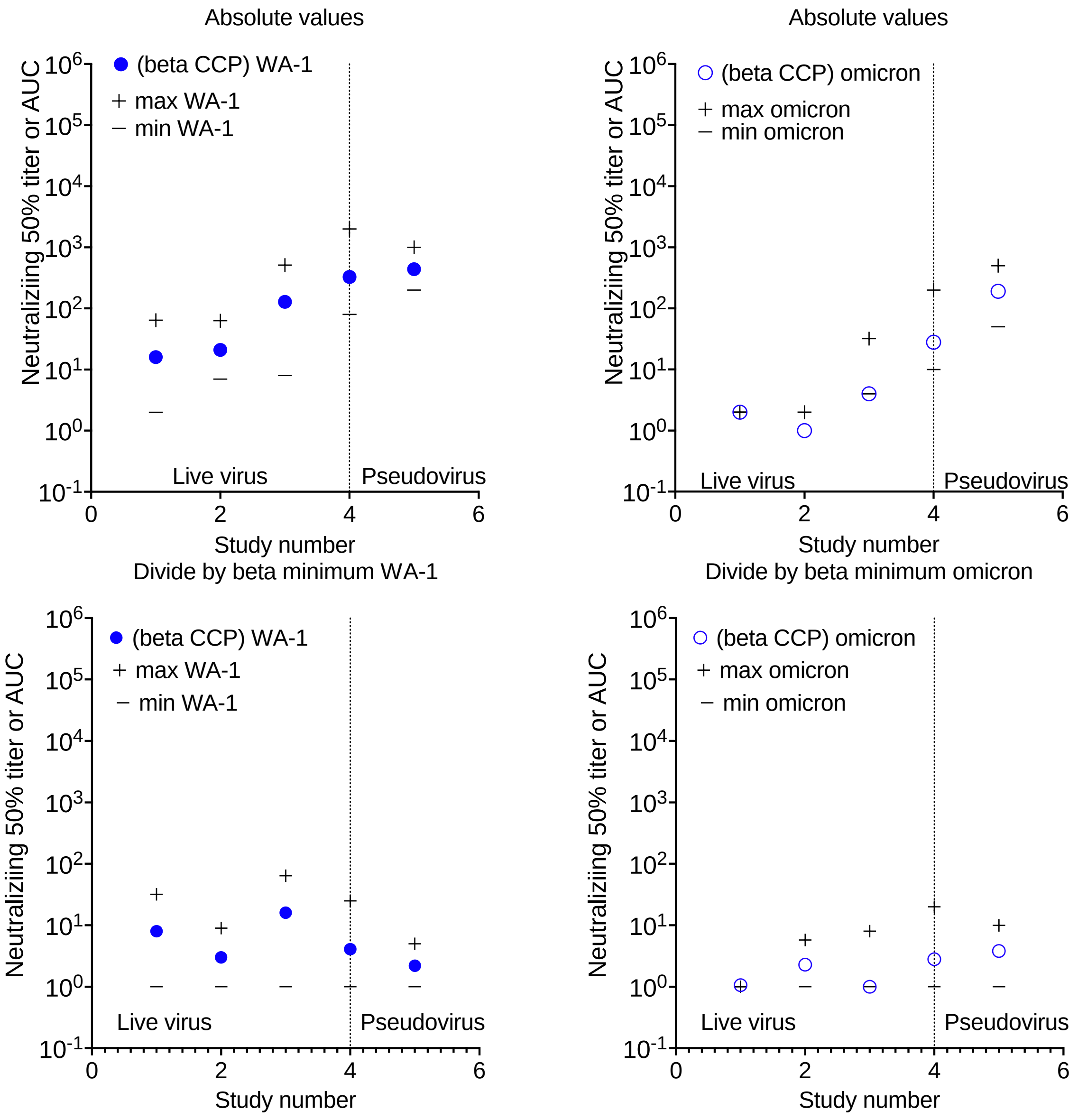

# Supplementary Figure 4

Alternative graphing of Supplementary table 4 and Figure 2A. 7 *in vitro* studies investigating the efficacy of Delta CCP against Omicron with $GMT_{50}$ from each study with minimum and maximum dilution titer also shown. The absolute values are graphed in the upper panels for WA-1 on left and omicron on right. In the lower panels all numbers divided by the minimum value for each study to normalize for WA-1 on left and Omicron on right. The $GMT_{50}$ approximates 10 with this computational division. Live virus assays are on the left and pseudoviral assays on the right. Individual studies did not report $GMT_{50}$ standard deviation.

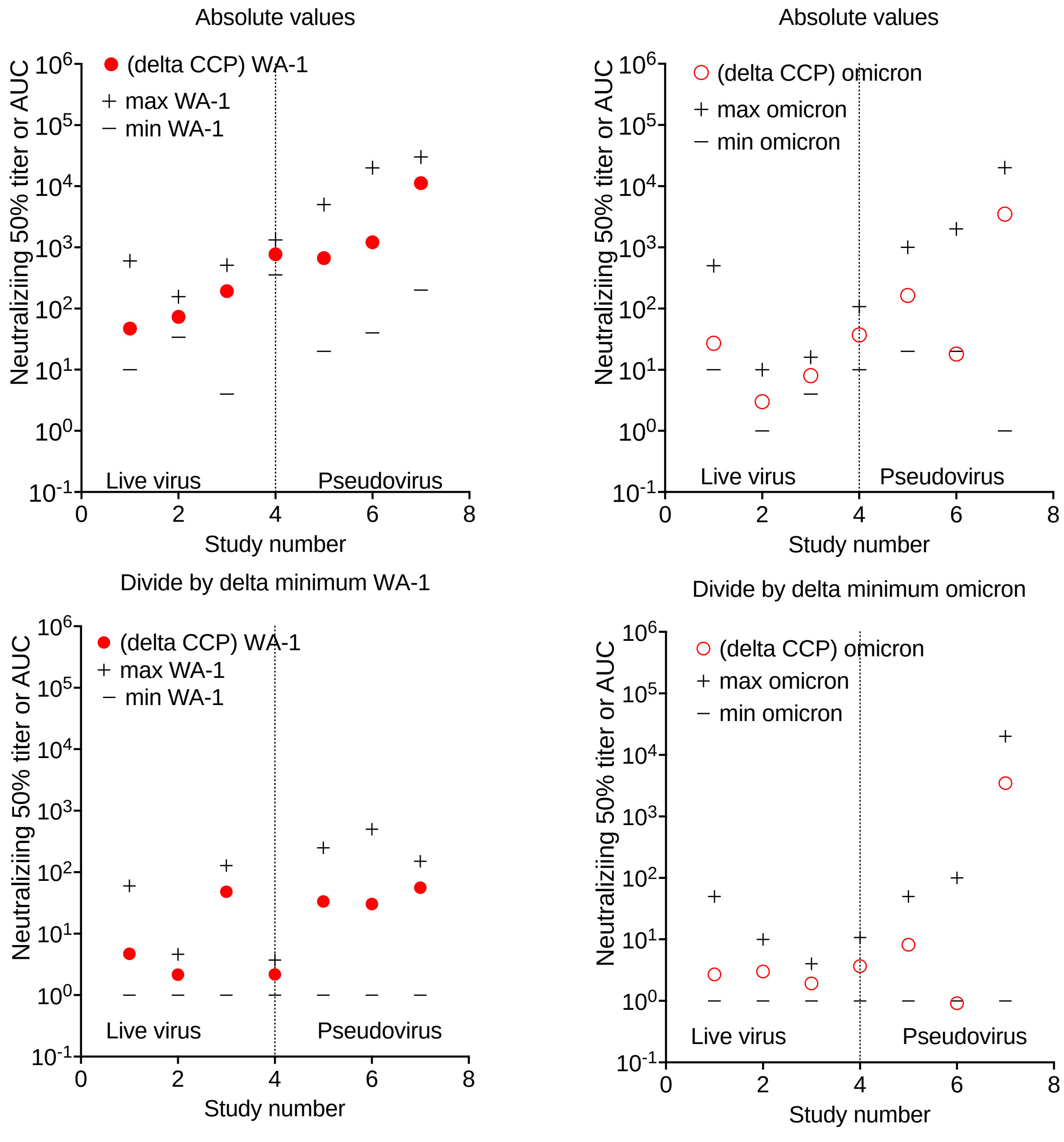

## Supplementary Figure 5

Alternative graphing of Supplementary table 5 and Figure 2B. 22 *in vitro* studies investigating the efficacy of plasma from uninfected recipients of 2 BNT162b2 doses against Omicron with $GMT_{50}$ from each study with minimum and maximum dilution titer also shown. The absolute values are graphed in the upper panels for WA-1 on left and omicron on right. In the lower panels all numbers divided by the minimum value for each study to normalize for WA-1 on left and omicron on right. The $GMT_{50}$ approximates 10 with this computational division. Live virus assays are on the left and pseudoviral assays on the right. Individual studies did not report $GMT_{50}$ standard deviation.

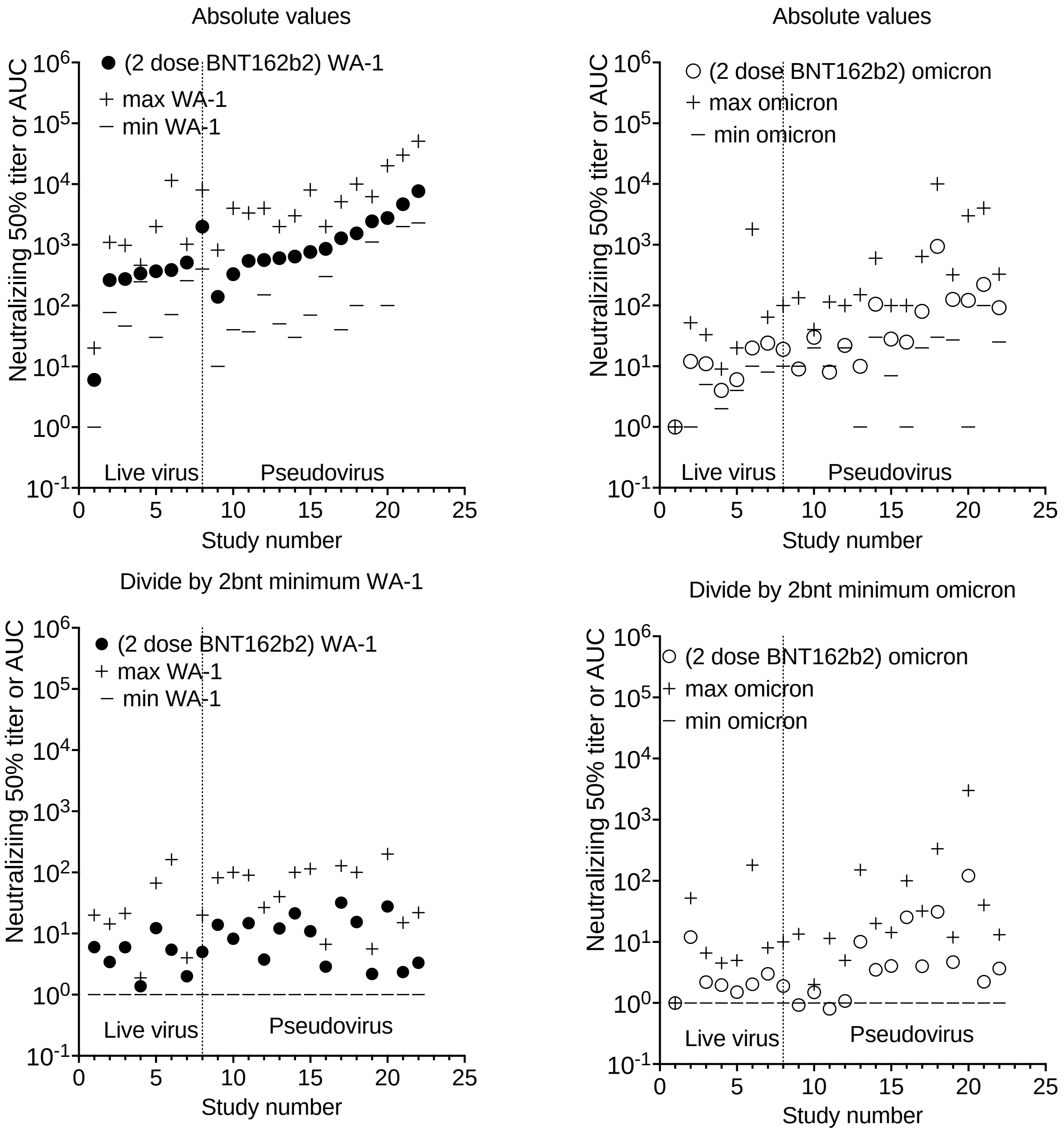

# Supplementary Figure 6

Alternative graphing of Supplementary table 6 and Figure 2B. 9 *in vitro* studies investigating the efficacy of plasma from uninfected recipients of 2 mRNA-1273 doses against Omicron with $GMT_{50}$ from each study with minimum and maximum dilution titer also shown. The absolute values are graphed in the upper panels for WA-1 on left and omicron on right. In the lower panels all numbers divided by the minimum value for each study to normalize for WA-1 on left and omicron on right. The $GMT_{50}$ approximates 10 with this computational division. Live virus assays are on the left and pseudoviral assays on the right. Individual studies did not report $GMT_{50}$ standard deviation.

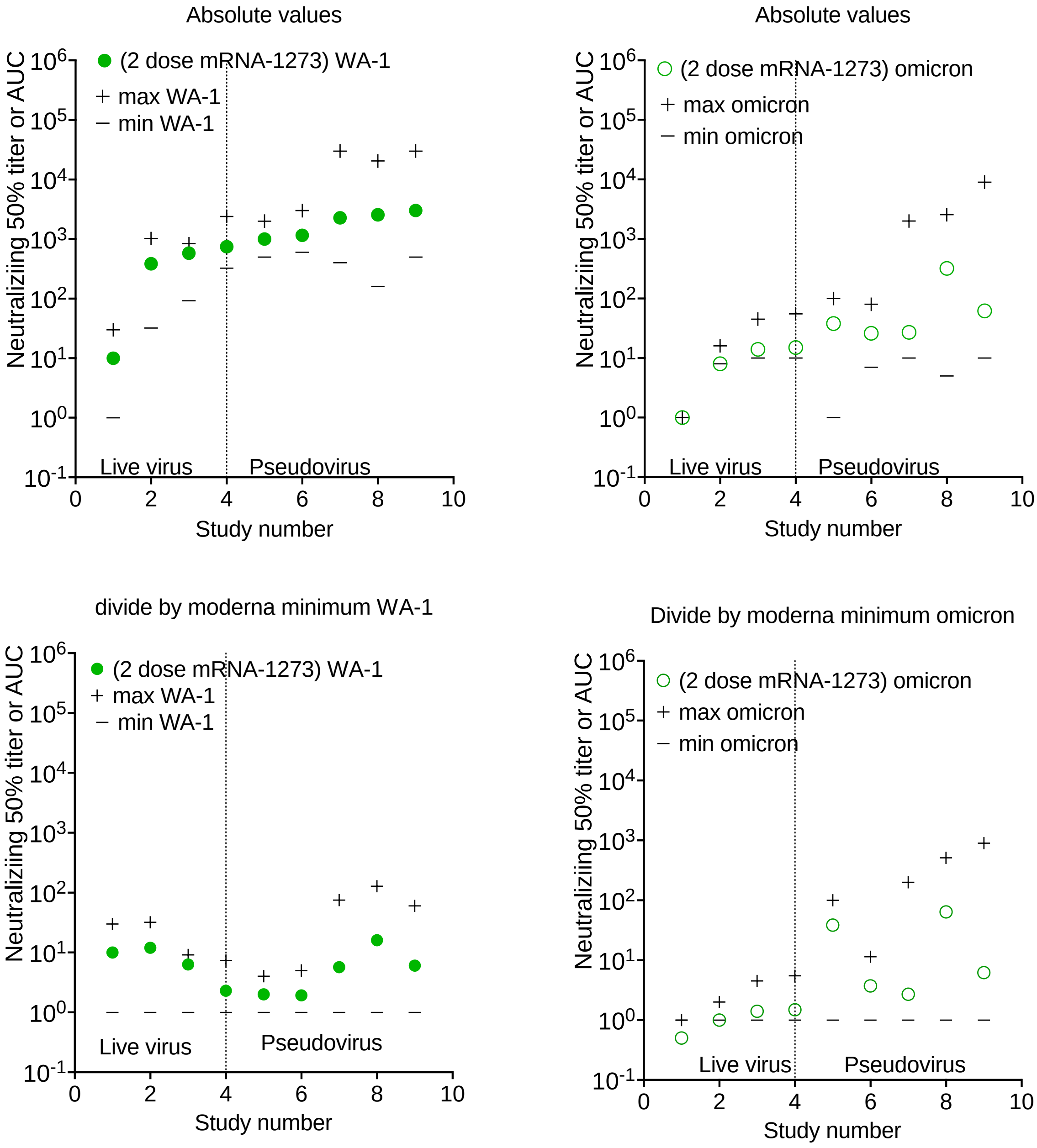

# Supplementary Figure 7

Alternative graphing of Supplementary table 7 and Figure 2B. 17 *in vitro* studies investigating the efficacy of plasma from infected and vaccinated (2 BNT162b2 doses) subjects (Vax-CCP) against Omicron with $GMT_{50}$ from each study with minimum and maximum dilution titer also shown. The absolute values are graphed in the upper panels for WA-1 on left and omicron on right. In the lower panels all numbers divided by the minimum value for each study to normalize for WA-1 on left and Omicron on right. The $GMT_{50}$ approximates 10 with this computational division. Live virus assays are on the left and pseudoviral assays on the right. Individual studies did not report $GMT_{50}$ standard deviation.

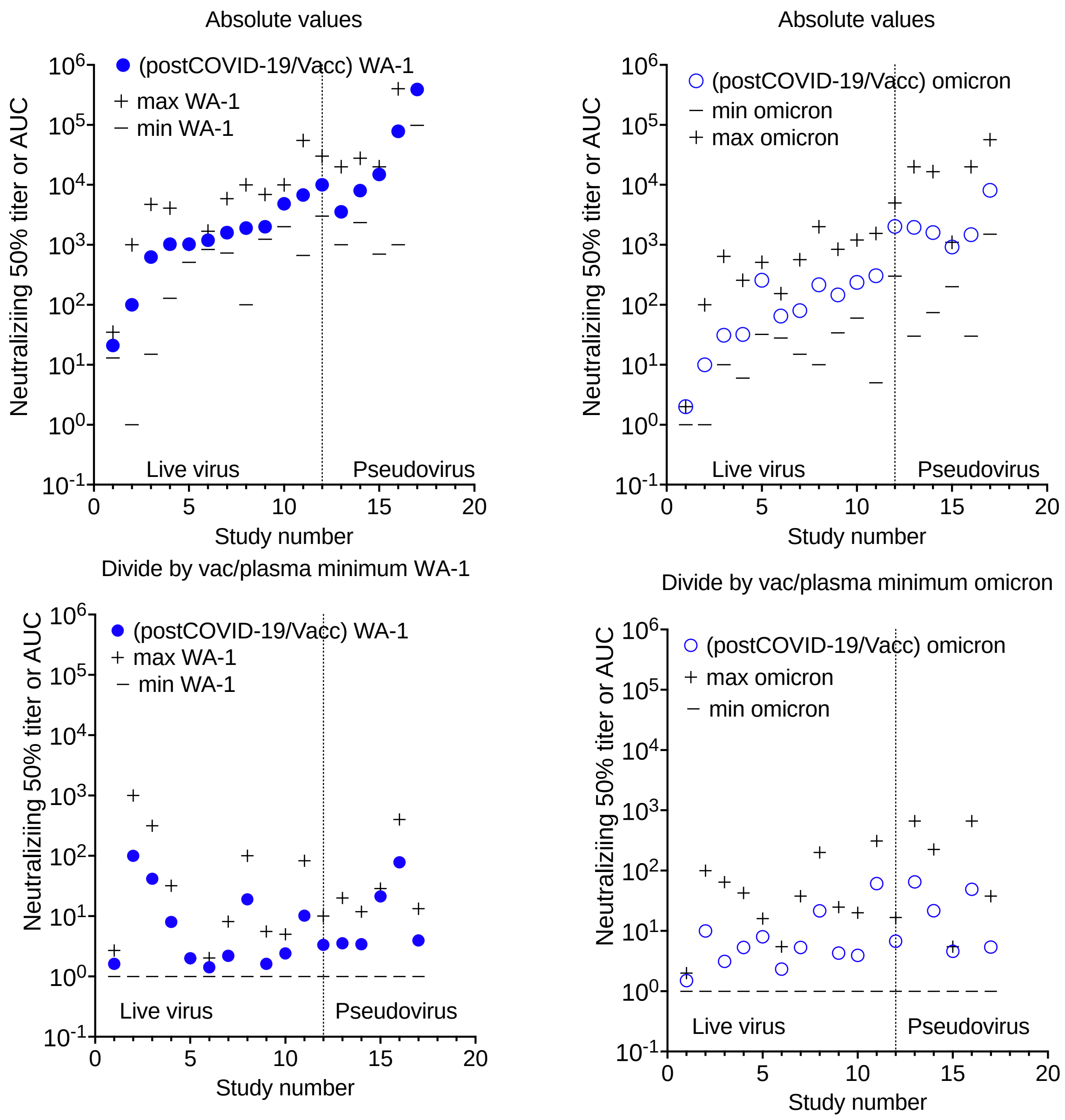

# Supplementary Figure 8

Alternative graphing of Supplementary table 8 and Figure 2B. 17 *in vitro* studies investigating the efficacy of plasma from uninfected subjects vaccinated with 3 BNT162b2 doses against Omicron with $GMT_{50}$ from each study with minimum and maximum dilution titer also shown. The absolute values are graphed in the upper panels for WA-1 on left and omicron on right. In the lower panels all numbers divided by the minimum value for each study to normalize for WA-1 on left and Omicron on right. The $GMT_{50}$ approximates 10 with this computational division. Live virus assays are on the left and pseudoviral assays on the right. Individual studies did not report $GMT_{50}$ standard deviation.

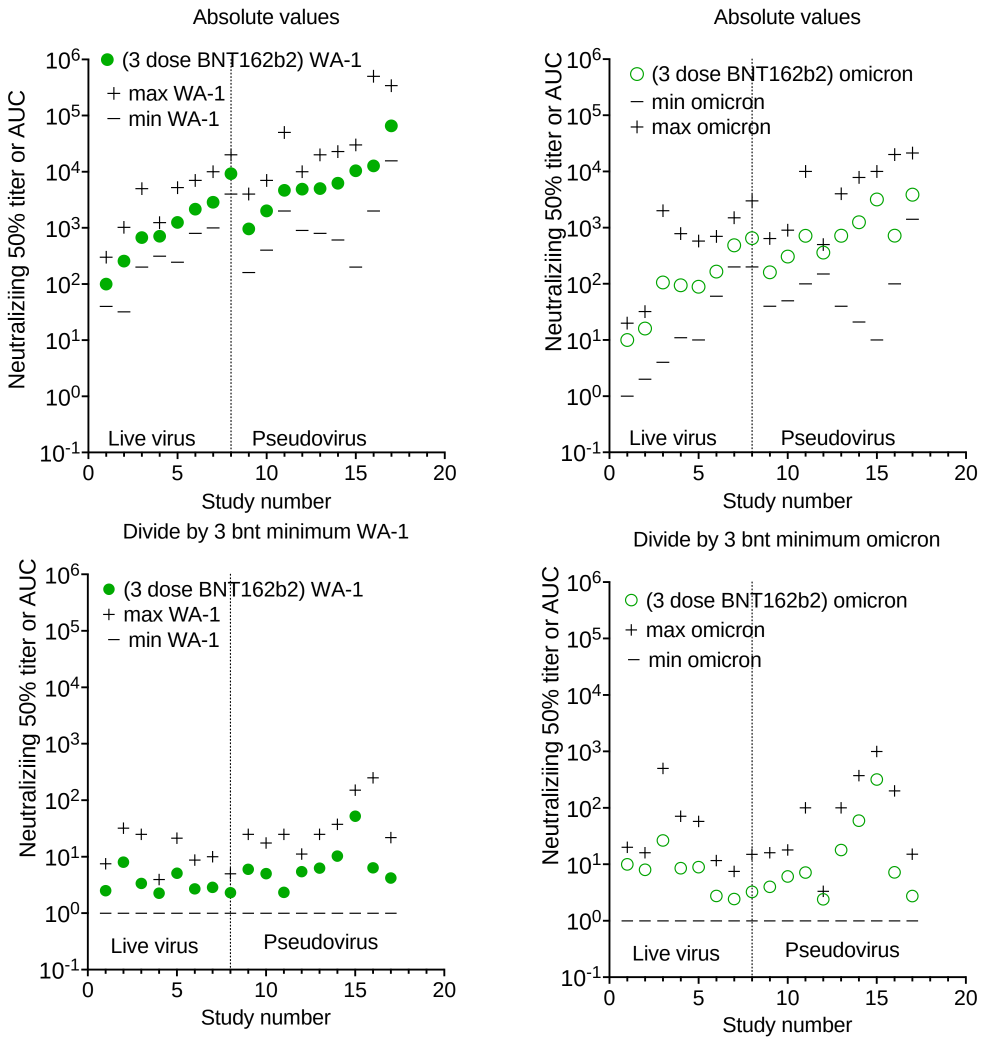

# Supplementary Figure 9

Alternative graphing of Supplementary table 10 and Figure 5. 7 *in vitro* studies investigating the efficacy of plasma from (pre-omicron, BA.1 and BA.4/5 CCP) subjects (Vax-CCP) against Omicron BA.1, BA.2 and BA.4/5 with $GMT_{50}$ from each study with minimum and maximum dilution titer also shown. The absolute values are graphed in the upper panels for WA-1 on left and omicron on right. In the lower panels all numbers divided by the minimum value for each study to normalize for WA-1 on left and Omicron on right. The $GMT_{50}$ approximates 10 with this computational division. Live virus assays are on the left and pseudoviral assays on the right. Individual studies did not report $GMT_{50}$ standard deviation.

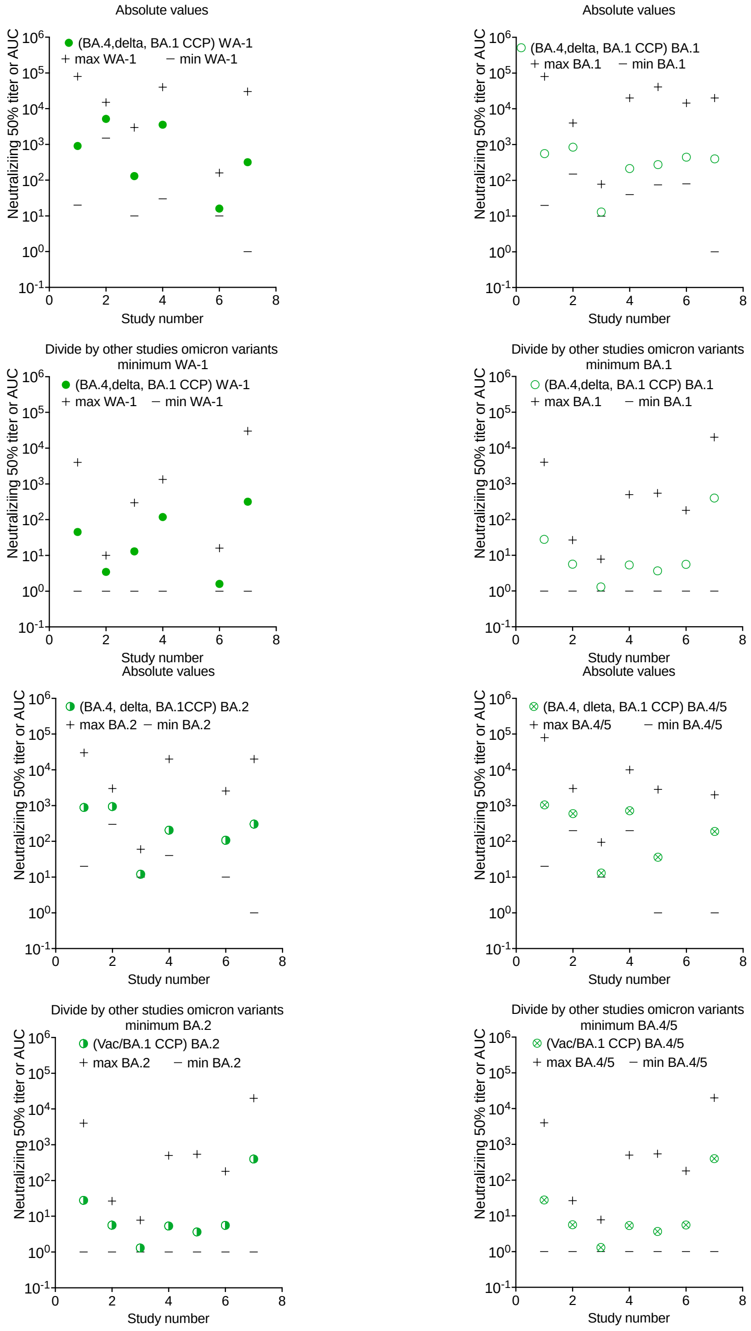

# Supplementary Figure 10

Alternative graphing of Supplement table 11 and Figure 5. 9 *in vitro* studies investigating the efficacy of plasma from (post-COVID-19-BA.1/full vacc plasma) subjects (Vax-CCP) against Omicron BA.1, BA.2 and BA.4/5 with $GMT_{50}$ from each study with minimum and maximum dilution titer also shown. The absolute values are graphed in the upper panels for WA-1 on left and omicron on right. In the lower panels all numbers divided by the minimum value for each study to normalize for WA-1 on left and Omicron on right. The $GMT_{50}$ approximates 10 with this computational division. Live virus assays are on the left and pseudoviral assays on the right. Individual studies did not report $GMT_{50}$ standard deviation.

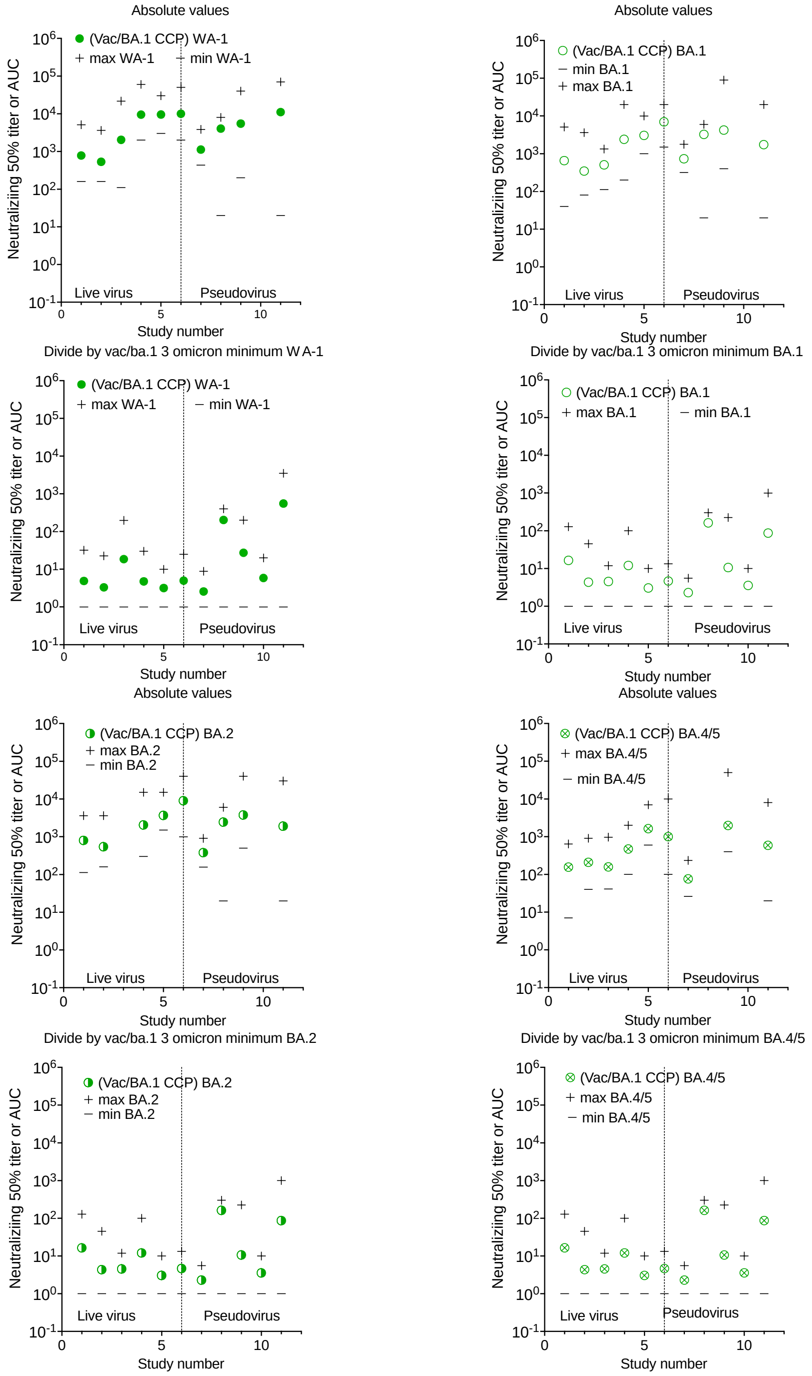

# Supplementary Figure 11

Alternative graphing of Supplementary table 12 and Figure 5. 8 *in vitro* studies investigating the efficacy of plasma from (3 dose BNT162b2 plasma) subjects (Vax-CCP) against Omicron BA.1, BA.2 and BA.4/5 with $GMT_{50}$ from each study with minimum and maximum dilution titer also shown. The absolute values are graphed in the upper panels for WA-1 on left and omicron on right. In the lower panels all numbers divided by the minimum value for each study to normalize for WA-1 on left and Omicron on right. The $GMT_{50}$ approximates 10 with this computational division. Live virus assays are on the left and pseudoviral assays on the right. Individual studies did not report $GMT_{50}$ standard deviation.

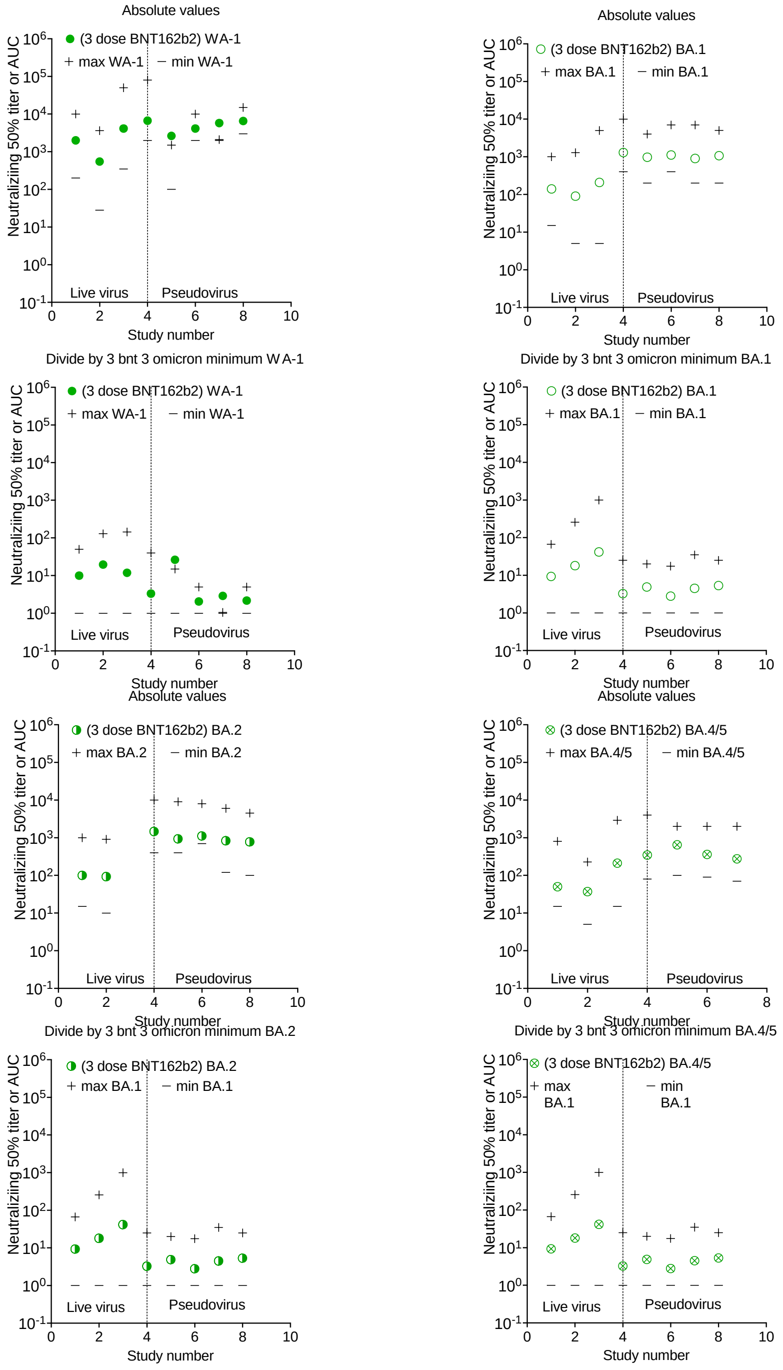

# References


1. Zeng C, *et al.* Neutralization and Stability of SARS-CoV-2 Omicron Variant. *bioRxiv*, (2021).

2. Liu L, *et al.* Striking antibody evasion manifested by the Omicron variant of SARS-CoV-2. *Nature* **602**, 676-681 (2022).

3. Schmidt F, *et al.* Plasma Neutralization of the SARS-CoV-2 Omicron Variant. *N Engl J Med* **386**, 599-601 (2022).

4. Arien KK, *et al.* Three doses of BNT162b2 vaccine confer neutralising antibody capacity against the SARS-CoV-2 Omicron variant. *NPJ Vaccines* **7**, 35 (2022).

5. Lusvarghi S, *et al.* SARS-CoV-2 BA.1 variant is neutralized by vaccine booster-elicited serum, but evades most convalescent serum and therapeutic antibodies. *Sci Transl Med*, eabn8543 (2022).

6. Hoffmann M, *et al.* The Omicron variant is highly resistant against antibody-mediated neutralization: Implications for control of the COVID-19 pandemic. *Cell* **185**, 447-456 e411 (2022).

7. Zou J, *et al.* Neutralization against Omicron SARS-CoV-2 from previous non-Omicron infection. *Nat Commun* **13**, 852 (2022).

8. Planas D, *et al.* Considerable escape of SARS-CoV-2 Omicron to antibody neutralization. *Nature* **602**, 671-675 (2022).

9. Zhang L, *et al.* The significant immune escape of pseudotyped SARS-CoV-2 variant Omicron. *Emerg Microbes Infect* **11**, 1-5 (2022).

10. Gruell H, *et al.* mRNA booster immunization elicits potent neutralizing serum activity against the SARS-CoV-2 Omicron variant. *Nat Med* **28**, 477-480 (2022).

11. Dejnirattisai W, *et al.* SARS-CoV-2 Omicron-B.1.1.529 leads to widespread escape from neutralizing antibody responses. *Cell* **185**, 467-484 e415 (2022).

12. Sheward DJ, *et al.* Variable loss of antibody potency against SARS-CoV-2 B.1.1.529 (Omicron). *bioRxiv*, 2021.2012.2019.473354 (2021).

13. Tada T, *et al.* Increased resistance of SARS-CoV-2 Omicron variant to neutralization by vaccine-elicited and therapeutic antibodies. *EBioMedicine* **78**, 103944 (2022).

14. Aggarwal A*, et al.* SARS-CoV-2 Omicron: evasion of potent humoral responses and resistance to clinical immunotherapeutics relative to viral variants of concern. *medRxiv*, 2021.2012.2014.21267772 (2021).

15. Zhao X*, et al.* Effects of a Prolonged Booster Interval on Neutralization of Omicron Variant. *N Engl J Med* **386**, 894-896 (2022).

16. Bowen JE*, et al.* Omicron BA.1 and BA.2 neutralizing activity elicited by a comprehensive panel of human vaccines. *bioRxiv*, (2022).

17. Carreno JM*, et al.* Activity of convalescent and vaccine serum against SARS-CoV-2 Omicron. *Nature* **602**, 682-688 (2022).

18. Syed AM*, et al.* Omicron mutations enhance infectivity and reduce antibody neutralization of SARS-CoV-2 virus-like particles. *medRxiv*, (2022).

19. Bekliz M*, et al.* Neutralization of ancestral SARS-CoV-2 and variants Alpha, Beta, Gamma, Delta, Zeta and Omicron by mRNA vaccination and infection-derived immunity through homologous and heterologous variants. 2021.2012.2028.21268491 (2021).

20. Haveri A*, et al.* Neutralizing antibodies to SARS-CoV-2 Omicron variant after third mRNA vaccination in health care workers and elderly subjects. *Eur J Immunol*, (2022).

21. Li M*, et al.* Convalescent plasma with a high level of virus-specific antibody effectively neutralizes SARS-CoV-2 variants of concern. *Blood advances*, 2022.2003.2001.22271662 (2022).

22. Kurahashi Y*, et al.* Cross-neutralizing activity against Omicron could be obtained in SARS-CoV-2 convalescent patients who received two doses of mRNA vaccination. *medRxiv*, 2022.2002.2024.22271262 (2022).

23. Rossler A, Riepler L, Bante D, von Laer D, Kimpel J. SARS-CoV-2 Omicron Variant Neutralization in Serum from Vaccinated and Convalescent Persons. *N Engl J Med* **386**, 698-700 (2022).

24. Lechmere T*, et al.* Broad Neutralization of SARS-CoV-2 Variants, Including Omicron, following Breakthrough Infection with Delta in COVID-19-Vaccinated Individuals. *mBio* **13**, e0379821 (2022).

25. Chatterjee D*, et al.* SARS-CoV-2 Omicron Spike recognition by plasma from individuals receiving BNT162b2 mRNA vaccination with a 16-week interval between doses. *Cell Rep* **38**, 110429 (2022).

26. Edara VV*, et al.* mRNA-1273 and BNT162b2 mRNA vaccines have reduced neutralizing activity against the SARS-CoV-2 omicron variant. *Cell Rep Med* **3**, 100529 (2022).

27. Muik A*, et al.* Neutralization of SARS-CoV-2 Omicron by BNT162b2 mRNA vaccine-elicited human sera. *Science* **375**, 678-680 (2022).

28. Cele S, *et al.* Omicron extensively but incompletely escapes Pfizer BNT162b2 neutralization. *Nature* **602**, 654-656 (2022).

29. Wilhelm A, *et al.* Reduced Neutralization of SARS-CoV-2 Omicron Variant by Vaccine Sera and Monoclonal Antibodies. *medRxiv*, 2021.2012.2007.21267432 (2021).

30. Doria-Rose NA, *et al.* Booster of mRNA-1273 Strengthens SARS-CoV-2 Omicron Neutralization. *medRxiv*, 2021.2012.2015.21267805 (2021).

31. Kawaoka Y, *et al.* Characterization and antiviral susceptibility of SARS-CoV-2 Omicron/BA.2. *Res Sq*, (2022).

32. Dejnirattisai W, *et al.* Reduced neutralisation of SARS-COV-2 Omicron-B.1.1.529 variant by post-immunisation serum. *Lancet* **399**, 234-236 (2022).

33. Wang Q, *et al.* Antibody evasion by SARS-CoV-2 Omicron subvariants BA.2.12.1, BA.4 and BA.5. *Nature*, (2022).

34. Awasthi M, Golding H, Khurana S. SARS-CoV-2 hyperimmune intravenous human immunoglobulins neutralizes Omicron subvariants BA.1, BA.2, BA.2.12.1, BA.3 and BA.4/BA.5 for treatment of COVID-19. *Clinical Infectious Diseases*, (2022).

35. Qu P, *et al.* Neutralization of the SARS-CoV-2 Omicron BA.4/5 and BA.2.12.1 Subvariants. *N Engl J Med* **386**, 2526-2528 (2022).

36. Khan K, *et al.* Omicron BA.4/BA.5 escape neutralizing immunity elicited by BA.1 infection. *Nat Commun* **13**, 4686 (2022).

37. Richardson SI, *et al.* SARS-CoV-2 BA.4 infection triggers more cross-reactive neutralizing antibodies than BA.1. 2022.2007.2014.500039 (2022).

38. Muik A, *et al.* Omicron BA.2 breakthrough infection enhances cross-neutralization of BA.2.12.1 and BA.4/BA.5. 2022.2008.2002.502461 (2022).

39. Planas D, *et al.* Duration of BA.5 neutralization in sera and nasal swabs from SARS-CoV-2 vaccinated individuals, with or without Omicron breakthrough infection. 2022.2007.2022.22277885 (2022).

40. Yu J, *et al.* Comparable Neutralization of the SARS-CoV-2 Omicron BA.1 and BA.2 Variants. 2022.2002.2006.22270533 (2022).

41. Tuekprakhon A, *et al.* Antibody escape of SARS-CoV-2 Omicron BA.4 and BA.5 from vaccine and BA.1 serum. *Cell* **185**, 2422-2433.e2413 (2022).

42. Hachmann NP*, et al.* Neutralization Escape by the SARS-CoV-2 Omicron Variants BA.2.12.1 and BA.4/BA.5. *N Engl J Med* **387**, 86-88 (2022).
